# A High-Performance Mn/TiO_2_ Catalyst with a High Solid Content for Selective Catalytic Reduction of NO at Low-Temperatures

**DOI:** 10.3390/molecules29153467

**Published:** 2024-07-24

**Authors:** Lei Yang, Zhen Wang, Bing Xu, Jie Hu, Dehua Pan, Guozhi Fan, Lei Zhang, Ziyang Zhou

**Affiliations:** Hubei Provincial Engineering Technology Research Center of Agricultural and Sideline Resources, Chemical Engineering and Utilization, School of Chemistry and Environmental Engineering, Wuhan Polytechnic University, Wuhan 430023, China; 17762666922@163.com (L.Y.); wz45151204@163.com (Z.W.); hujie9231@126.com (J.H.); 17362455864@163.com (D.P.); fgzcch@whpu.edu.cn (G.F.); zhanglei@whpu.edu.cn (L.Z.); 13257115373@163.com (Z.Z.)

**Keywords:** NH_3_-SCR reaction, low temperature, different solid contents, Mn/TiO_2_ catalysts, degradation of NO

## Abstract

Mn/TiO_2_ catalysts with varying solid contents were innovatively prepared by the sol–gel method and were used for selective catalytic reduction of NO at low temperatures using NH_3_ (NH_3_-SCR) as the reducing agent. Surprisingly, it was found that as the solid content of the sol increased, the catalytic activity of the developed Mn/TiO_2_ catalyst gradually increased, showing excellent catalytic performance. Notably, the Mn/TiO_2_ (50%) catalyst demonstrates outstanding denitration performance, achieving a 96% NO conversion rate at 100 °C under a volume hourly space velocity (VHSV) of 24,000 h^−1^, while maintaining high N_2_ selectivity and stability. It was discovered that as the solid content increased, the catalyst’s specific surface area (SSA), surface Mn^4+^ concentration, chemisorbed oxygen, chemisorption of NH_3_, and catalytic reducibility all improved, thereby enhancing the catalytic efficiency of NH_3_-SCR in degrading NO. Moreover, NH_3_ at the Lewis acidic sites and NH^4+^ at the Bronsted acidic sites of the catalyst were capable of reacting with NO. Conversely, NO and NO_2_ adsorbed on the catalyst, along with bidentate and monodentate nitrates, were unable to react with NH_3_ at low temperatures. Consequently, the developed catalyst’s low-temperature catalytic reaction mechanism aligns with the E-R mechanism.

## 1. Introduction

In recent years, as industry has rapidly developed, environmental pollution has become a pressing issue. Industrial NO_x_ emissions cause significant environmental damage, including acid rain, the greenhouse effect, and haze. Additionally, the high mobility of NO_x_ compounds can easily lead to global issues [1,2,3,4]. To the human body, NO_x_ is one of the causes of respiratory diseases, posing a serious threat to personal safety [5]. The issue of NO_x_ emissions has garnered widespread attention across countries. Developing technologies that can effectively handle NO_x_ has become essential for addressing air pollution.

Currently, the NH_3_-SCR technique is the most widely applied mature flue gas denitrification technology, with its core being the selective catalyst [6]. The traditional commercial V_2_O_5_-WO_3_(MoO_3_)/TiO_2_ catalyst exhibits its highest activity within the temperature range of 250–400 °C achieving a NO_x_ conversion rate of up to 90% [7]. However, the catalytic activity drops significantly when the flue gas temperature falls below 200 °C.

Recent research indicates that manganese-based catalysts have exhibited exceptional low-temperature catalytic activity. This is primarily attributed to the high concentration of active oxygen species within these catalysts and the numerous acidic sites present on the MnO_x_ surface, enabling them to efficiently facilitate the NH_3_-SCR reaction even at low temperatures [8,9]. TiO_2_ boasts a large specific surface area, endowing it with robust adsorption and loading capabilities. The loading capacity influences the denitrification efficiency, making it a favored choice as the support for denitrification catalysts [10]. The Mn/TiO_2_ catalyst exhibits outstanding catalytic performance at low temperatures, positioning it as a focal point in the research of low-temperature denitrification techniques [11,12].

Research has found that compared with traditional MnO_x_-TiO_2_ catalysts, the incorporation of metal elements such as Ce, Fe, Pb, Sb, Sm, and Pr, along with the preparation of composite supports, can significantly enhance the catalyst’s low-temperature catalytic performance [13,14,15,16,17,18,19,20]. Wu et al. [21] synthesized a series of Mn/TiO_2_ catalysts modified by doping with different transition metals (Fe, Cu, Ni, Cr) using the sol–gel method. Among them, the Fe_0.1_Mn_0.4_/TiO_2_ catalyst has the highest catalytic activity. Lu et al. [22] first prepared the TiO_2_Gr composite nanocarrier using the sol–gel method, and then loaded the Mn and Ce active components onto the composite nanocarrier using the impregnation method, synthesizing a series of CeMn/TiO_2_Gr catalysts. When the Ce/Mn molar ratio is 0.3 and the mass fraction of Ce and Mn is 7%, the catalyst exhibits the best catalytic performance. Chen et al. [23] synthesized a series of Co_0.2_CexMn_0.8-x_Ti_10_ catalysts by the sol–gel method. The results showed that the Co_0.2_Ce_0.35_Mn_0.45_Ti_10_ sample had the best NH_3_-SCR activity. Co and Ce doping can provide more acid sites and NO adsorption sites, further improving the catalytic activity of the catalyst.

Currently, there are many reports in the literature about catalysts prepared by the sol–gel method, but there is less research on the impact of changes in sol solid content on the catalytic performance of the catalysts. This paper mainly uses the sol–gel method to prepare a series of Mn/TiO_2_ catalysts with varying solid contents, investigating the mechanism linking the impact of solid content variations on catalyst structure and performance, and further explores the reaction mechanism of Mn/TiO_2_ (50%) catalyst sample NH_3_-SCR degradation of NO at low temperatures.

## 2. Results and Discussion

### 2.1. Catalytic Performance of Catalysts

**Figure 1:**
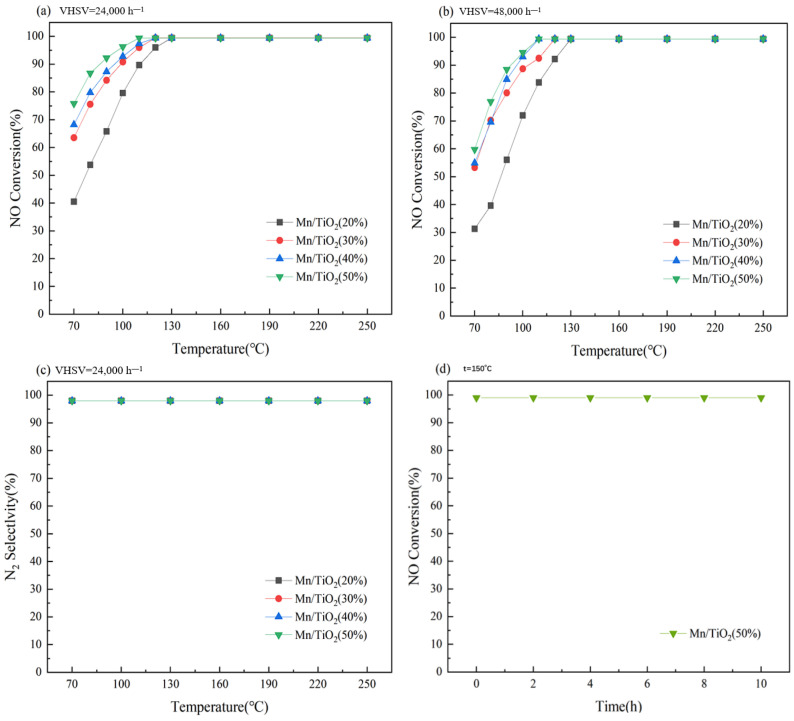
NO conversion rates (**a**,**b**) of catalysts at VHSV of 24,000 h^−1^ and 48,000 h^−1^; N_2_ selectivity (**c**) and stability (**d**) at 24,000 h^−1^.

To investigate the NH_3_-SCR catalytic efficacy of the catalysts, the catalyst’s activity was assessed via a reactor setup. Figure 1a illustrates the variations in NO conversion rates for various catalysts at a VHSV of 24,000 h^−1^. The NO conversion rate of each catalyst showed good catalytic performance in the temperature range of 70–130 °C, and the conversion rate gradually increased with the increase in solid content, indicating that the reaction efficiency gradually improved. It is speculated that this is mainly due to the increase in solid content, such that the specific surface area of the catalyst tends to increase. The Mn/TiO_2_ (50%) catalyst achieved a NO conversion rate of 96% at 100 °C and sustained a rate of 99% across the temperature range of 110–250 °C. Figure 1b shows the changes in NO conversion rates of various catalysts at a VHSV of 48,000 h^−1^. The catalytic activity of the catalysts has slightly decreased, but the NO conversion rate of the Mn/TiO_2_ (50%) catalyst remains the highest, reaching 99% even after 110 °C. Therefore, it can be predicted that the Mn/TiO_2_ (50%) catalyst has a higher contact surface area, resulting in higher catalytic reaction efficiency. As shown in Figure 1c, the N_2_ selectivity of all the catalysts remains above 98%. Figure 1d depicts the stability test curve for the Mn/TiO_2_ (50%) catalyst at 150 °C. The graph demonstrates that the catalyst maintains excellent stability for 10 h. In order to further clarify the mechanism and reasons for the impact of increased solid content on the catalytic performance of catalysts, a series of characterization analyses were subsequently conducted.

### 2.2. Morphology, Crystallinity, and Porous Property Analysis

**Figure 2:**
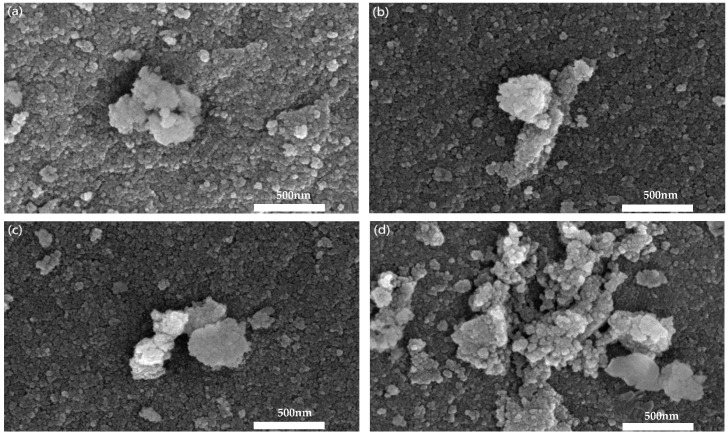
SEM images of catalysts: (**a**) Mn/TiO_2_ (20%), (**b**) Mn/TiO_2_ (30%), (**c**) Mn/TiO_2_ (40%), (**d**) Mn/TiO_2_ (50%).

To observe the impact of varying solid contents on the surface micromorphology of Mn/TiO_2_ catalysts, SEM observations were conducted on catalysts. Figure 2 displays the SEM images of catalysts. The particles on the surface of the catalyst are spherical, with uniform size and local agglomeration (with Mn/TiO_2_ (50%) being more obvious), but the overall dispersion is good, indicating that catalysts with different solid contents have similar microstructures on their surfaces.

**Figure 3:**
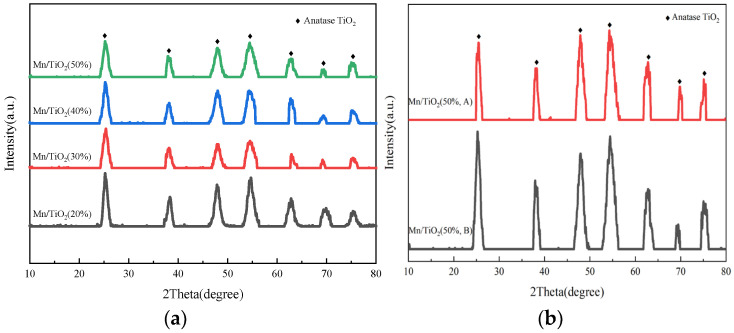
XRD patterns of different solid contents catalysts (**a**) and Mn/TiO_2_ (50%) catalysts before (B) and after (A) stability testing (**b**).

Figure 3a displays the XRD patterns of Mn/TiO_2_ catalysts with varying solid contents. The figures reveal that all catalysts display similar characteristic XRD diffraction peaks, and the solid content variations do not alter the crystal forms of the catalysts. Diffraction peaks near 2θ = 25.2°, 38.0°, 47.9°, 54.4°, 62.8°, 69.1°, and 75.1° are characteristic of anatase TiO_2_, which is conducive to enhanced low-temperature SCR catalytic performance. No characteristic peaks of manganese oxides were detected on the surfaces of the catalysts, suggesting that manganese oxides are either highly evenly dispersed on the catalyst surfaces, exist in an amorphous state, or are present in concentrations too low to be detected by XRD [24]. The XRD analysis indicates that the diffraction peak intensity of Mn/TiO_2_ (20%) is the highest. As the solid content of Mn/TiO_2_ increases, the intensity of the characteristic peaks diminishes, and the crystallinity of the TiO_2_ support decreases, The change in solid content has an impact on the diffraction peak of the titanium dioxide carrier. As depicted in Figure 3b, even after the catalyst stability test, similar peaks are still present; the peak at 2θ = 25.2° weakens slightly, suggesting that the catalyst’s structure remains largely unchanged after the stability test. At the same time, XRF tests were conducted on the active metal loading before and after the stability test of the catalyst. The Mn content of the active metal before the catalyst reaction was 19.50%, and the content remained at 19.22% after the stability test. Therefore, it once again strongly supports the conclusion of stability testing.

**Figure 4:**
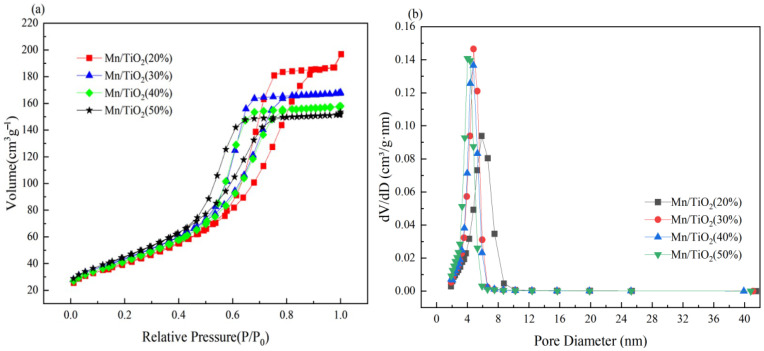
N_2_ adsorption–desorption diagram (**a**) and pore size distribution curve (**b**) of catalysts.

Figure 4 shows the adsorption–desorption isotherm and pore size distribution curve of the catalysts. As illustrated in Figure 4a, the adsorption–desorption isotherms of catalysts display characteristic IV and H 2 type hysteresis loops, confirming that all catalysts possess mesoporous structures [25,26]. Figure 4b presents the pore structure distribution curve. As depicted, the pore sizes of the catalysts primarily fall within the range of 2–10 nm. Table 1 provides the data related to the SSA and pore size of catalysts. The SSA and pore volume data were derived from BET and BJH analyses. As indicated in Table 1, with an increase in solid content, the SSA of the catalyst markedly increases from 146.44 m^2^/g to 165.34 m^2^/g. This suggests that an increase in solid content positively impacts the catalyst’s SSA. This likely stems from a more uniform distribution of active components on the catalyst surface, leading to smaller pore sizes and an increased number of pores with higher solid content. Among the catalysts, Mn/TiO_2_ (50%) exhibits the highest SSA, favoring an increase in the adsorbed amount and rate of reactants on the catalyst surface, and enhancing the desorption of reaction products, thus boosting the catalyst’s low-temperature SCR catalytic performance [27]. This validates the results of performance testing and predictive analysis very well.

### 2.3. XPS Analysis

**Figure 5:**
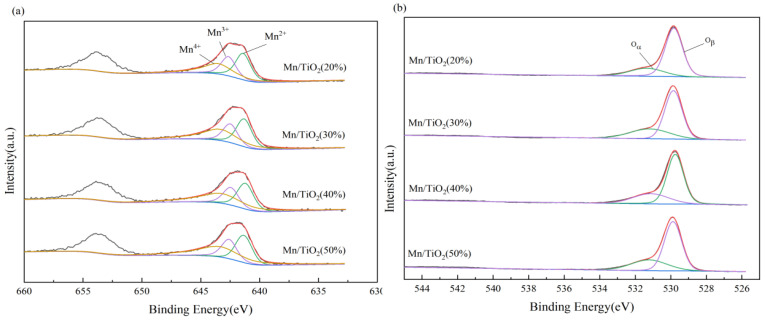
XPS profiles of catalysts: (**a**) Mn 2p, (**b**) O 1s.

To study the impact of valence changes of surface elements on the catalytic performance of catalysts, XPS measurements were conducted on all catalysts. As depicted in Figure 5a, the primary peaks of Mn 2p3/2 and Mn 2p1/2 are located at approximately 642.0 eV and 653.5 eV, respectively. Peak fitting of Mn 2p3/2 yielded three electronic peaks: Mn^2+^ (641.4 ± 0.1 eV), Mn^3+^ (642.6 ± 0.1 eV), and Mn^4+^ (643.6 ± 0.1 eV) [27]. As indicated in Table 2, as the solid content of the catalyst increases, the ratio of Mn^4+^/(Mn^2+^ + Mn^3+^ + Mn^4+^) progressively increases. Therefore, the Mn^4+^ content is highest for Mn/TiO_2_ (50%) catalysts. High concentration of Mn^4+^ enhances low-temperature SCR catalytic reduction reactions [24].

Figure 5b displays two overlapping peaks at 529.8 eV and 531.5 eV, resulting from the fitting of the O 1s peaks [28]. The peak at 529.8 eV corresponds to lattice oxygen (O_β_), whereas the peak at 531.5 eV corresponds to chemisorbed oxygen (O_α_). On the catalyst surface, O_α_ demonstrates a greater migration rate than O_β_. An elevated ratio of O_α_/(O_α_ + O_β_) improves the oxidation capability of NO at low temperatures and speeds up the SCR reaction during these conditions [24,25,26,27,28,29]. Table 2 demonstrates that Mn/TiO_2_ (50%) exhibits the highest Mn^4+^/Mn^n+^ and O_α_/(O_α_ + O_β_) ratios, suggesting that as the solid content increases, both the Mn^4+^ concentration and chemisorbed oxygen content on the catalyst surface rise, contributing to its superior catalytic performance at low temperatures. This conclusion once again strongly supports the performance testing results of the catalyst.

### 2.4. H_2_-TPR, NH_3_-TPD Analysis

**Figure 6:**
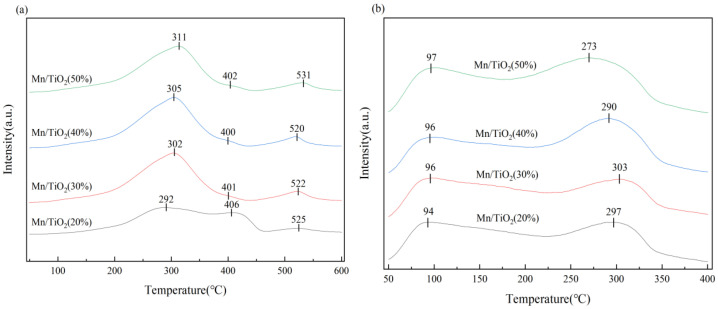
H_2_-TPR (**a**) and NH_3_-TPD (**b**) spectra of catalysts.

To explore the impact of varying solid contents on the redox performance of Mn/TiO_2_ catalysts, H_2_-TPR tests were conducted on catalysts. Figure 6a displays the H_2_-TPR spectra for catalysts, revealing three reduction peaks between 250–550 °C. The reduction peak around 300 °C is attributed to the transformation from MnO_2_ to Mn_2_O_3_, the peak near 400 °C to the transition from Mn_2_O_3_ to Mn_3_O_4_, and the peak near 520 °C to the reduction from Mn_3_O_4_ to MnO [30,31,32]. With the increase in solid content of the catalyst, the reduction peaks of the Mn/TiO_2_ catalysts generally shift towards higher temperatures. Calculations of the peak areas, as detailed in Table 2, reveal that the Mn/TiO_2_ (50%) catalyst exhibits the largest peak area, suggesting an abundance of reducing substances within the catalyst, therefore enhancing the low-temperature SCR reaction.

Studies have demonstrated that a positive correlation exists between the number of acidic sites on the surface of catalysts and their catalytic activity. Consequently, catalysts were subjected to NH_3_-TPD characterization. Figure 6b displays the NH_3_-TPD spectra for catalysts, revealing two distinct strong desorption peaks. Literature records indicate that the desorption peak near 100 °C is attributed to NH_3_ adsorbed on Bronsted acidic sites (weak acids) [33,34], while the peak near 300 °C corresponds to NH_3_ adsorbed on medium-strength acid sites [34]. The surface acidity of the catalyst was semi-quantitatively obtained by calculating the peak area of the NH_3_-TPD spectrum; as shown in Table 2, the Mn/TiO_2_ (50%) catalyst exhibited the largest peak area for NH_3_ desorption, indicating the highest number of acid sites on its surface. Additionally, the graph demonstrates that the Mn/TiO_2_ (50%) catalyst achieves its peak area at low temperatures ranging from 50 to 150 °C, correlating with its superior low-temperature catalytic activity, which aligns with performance test results.

### 2.5. In Situ DRIFTS

**Figure 7:**
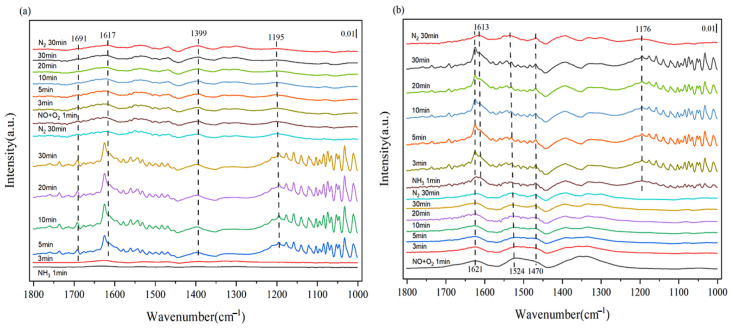
The in situ infrared spectrum of the NO + O_2_ (**a**) (NH_3_ (**b**)) reaction, following the pre-adsorption of NH_3_ (**a**) (NO + O_2_ (**b**)) on the Mn/TiO_2_ (50%) catalyst at 120 °C.

In order to illustrate the types of ammonia and NO formed by NH_3_-SCR and the reaction mechanism, in situ drift measurements were conducted on the Mn/TiO_2_ (50%) catalyst. Figure 7a presents the in situ infrared spectrum of the NO + O_2_ adsorption reaction over time, following the pre-adsorption of NH_3_ on the Mn/TiO_2_ (50%) catalyst at 120 °C. Upon introducing NH_3_ gas, the absorption peaks at 1195 cm^−1^ and 1617 cm^−1^ are attributed to the vibrations of NH_3_ species on Lewis acidic sites, while the characteristic peaks at 1399 cm^−1^ and 1691 cm^−1^ are due to NH^4+^ on Bronsted acidic sites. Upon introducing NO and O_2_ into the reaction tank, the peak corresponding to ammonia adsorption decreases. As the levels of NO and O_2_ in the reaction cells increase, the peak intensities of NH_3_ (1195 cm^−1^) on Lewis acidic sites and NH^4+^ (1691 cm^−1^) on Bronsted acidic sites gradually diminish. This experiment demonstrates that both NH_3_ on Lewis acidic sites and NH^4+^ on Bronsted acidic sites are capable of reacting with NO + O_2_.

**Figure 8:**
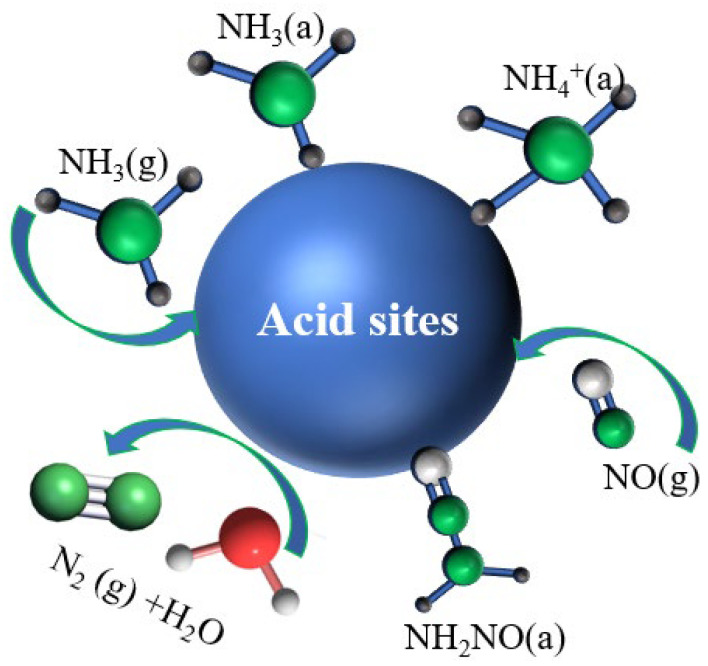
E-R reaction mechanism diagram.

Figure 7b presents the in situ infrared spectrum of the NH_3_ adsorption reaction over time, following the pre-adsorption of NO + O_2_ on the Mn/TiO_2_ (50%) catalyst at 120 °C. Upon introducing the NO + O_2_ gas, the absorption peak at 1621 cm^−1^ is attributed to the weak adsorption peaks of NO and NO_2_, the peak at 1524 cm^−1^ to bidentate nitrate, and the peak at 1470 cm^−1^ to monodentate nitrate. Upon switching to NH_3_, the intensities of the three absorption peaks remained nearly unchanged, with new characteristic peaks emerging at 1176 and 1613 cm^−1^, attributed to the characteristic peaks of ammonia species. NO and NO_2_ adsorbed on the catalyst, along with bidentate and monodentate nitrates, are unable to react with NH_3_ at low temperatures. As indicated in Figure 7a, the reaction mechanism of the Mn/TiO_2_ catalyst is determined to be the E-R mechanism (Figure 8) [28].

## 3. Experimental Section

### 3.1. Preparation of Catalysts

A series of Mn/TiO_2_ catalysts with varying solid contents were synthesized by the sol–gel method. The specific steps are as follows: first, put Ti[CH_3_(CH_2_)_3_O]_4_ (AR, Sinopharm Chemical, Shanghai, China) into a mixed solution containing CH_3_COOH (AR, Sinopharm Chemical), CH_3_CH_2_OH (AR, Sinopharm Chemical) and ultrapure water (UP, laboratory-made), then add Mn(NO_3_)_2_ (AR, 50 wt% in H_2_O, Shanghai MacLean reagent), stir vigorously at room temperature for 5 h, and then place in a drying oven at 30 °C for 144 h to obtain the gel. The solid obtained was dried at 60 °C for 8 h, then at 120 °C for 8 h, followed by calcination in air atmosphere at 400 °C for 4 h and sieving to produce a 40–60 mesh finished catalyst. The Mn/TiO_2_ catalysts had solid contents of 20%, 30%, 40%, and 50%, respectively, the Mn/Ti molar ratio was fixed at 0.3. The synthesized catalyst is denoted as Mn/TiO_2_ (x), where x indicates the solid content of the sol (x = (m_Ti[CH3(CH2)3O]4_ + m_Mn(NO3)2_)/(m_Ti[CH3(CH2)3O]4_ + m_Mn(NO3)2_ + m_solvent_), x varies with the change of m_solvent_). The loading amount of active metal remains constant.

### 3.2. Characterizations

Scanning electron microscopy (JSM-7610FPlus, JEOL, Tokyo, Japan) was employed to observe the microstructure and morphology of the surfaces of catalysts. The stability test of the catalyst before and after the active metal loading test was conducted using an X-ray diffraction spectrometer (EDX-7000, Shimadzu, Kagoshima, Japan). The specific surface area (SSA) and pore volume of catalysts were determined by the N_2_ adsorption–desorption principle (ASAP2460, Micromeritics, Norcross, GA, USA), and calculated using the Brunauer–Emmett-Teller (BET) and Barrett–Joyner–Halenda (BJH) methods. The crystal structure of catalysts was measured using X-ray diffraction (XRD-7000, Shimadzu, Kagoshima, Japan). X-ray photoelectron spectroscopy (Escalab 250xi, ThermoFisher, Waltham, MA, USA) was used to analyze the valence state and composition of the elements on the catalysts surface, with the Al K Alpha target serving as the excitation source, and the C1s peak (284.8 eV) employed to calibrate the elements for catalysts. H_2_-TPR and NH_3_-TPD were measured using a temperature programmed chemical adsorption analyzer TPD/TPR (AutoChem II 2920, Micromeritics, Norcross, GA, USA). Firstly, the catalyst sample was subjected to drying pretreatment at 300 °C, followed by N_2_ (30 mL/min) blowing for 1 h. Then, NH_3_ (or H_2_) was adsorbed for 1 h under 10% NH_3_/N_2_ (or 10% H_2_/N_2_). The Fourier Transform Infrared Spectrometer (INVENIO S, Bruker, Karlsruhe, Germany) was used for in situ diffuse reflectance infrared spectroscopy experiments. The catalyst sample was purged with N_2_ at 300 °C for 30 min, and then stabilized at 120 °C for subsequent measurements.

### 3.3. Performance Testing of Catalysts

**Figure 9:**
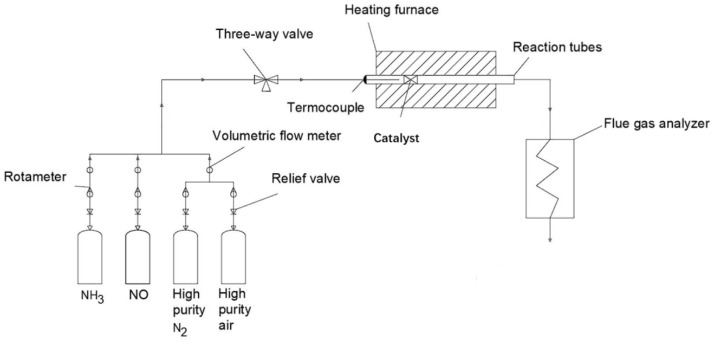
Flowchart of the experimental apparatus for catalyst NH_3_-SCR performance testing.

The denitrification performance of the catalyst was tested in a fixed-bed quartz tube reactor. As illustrated in Figure 9, the reaction gas comprises 0.6% NO, 0.3% NH_3_, high-purity O_2_, and high-purity N_2_, with respective concentrations of (350 ppm) NO, (350 ppm) NH_3_, 5% O_2_, and 5% N_2_ as the equilibrium gas. The catalyst (40–60 mesh), with a bed height of 1 cm (approximately 0.88 g), was placed at the temperature-controlled section of the reaction tube. The total gas flow rates were set at 300 mL/min and 600 mL/min, and the VHSV values were 24,000 h^−1^ and 48,000 h^−1^. An online gas analyzer (GW-2000) was used to measure the NO content in the tail gas following the reaction. A specific gas detection tube (MHY-15232) was used to measure the NH_3_ content at both the inlet and outlet and an N_2_O detector (FGD2-C-N2O) was employed to determine the N_2_O content generated in the reaction’s exhaust gas. The conversion rate of NO and the selectivity towards N_2_ were calculated using the following Formulas (1) and (2).

(1) $$NO\;conversion(\%)=\frac{{\left[NO\right]}_{in}-{\left[NO\right]}_{out}}{{\left[NO\right]}_{in}}\times 100\%$$

(2) $$N_{2}\;selectivity(\%)=\frac{{\left[NO\right]}_{in}+{\left[{NH}_{3}\right]}_{in}-{\left[NO\right]}_{out}-{\left[{NH}_{3}\right]}_{out}-{\left[{NO}_{2}\right]}_{out}-{2[N_{2}O]}_{out}}{{\left[NO\right]}_{in}+{\left[{NH}_{3}\right]}_{in}-{\left[NO\right]}_{out}-{\left[{NH}_{3}\right]}_{out}}\times 100\%$$

## 4. Conclusions

Mn/TiO_2_ catalysts with varying solid contents were innovatively prepared by the sol–gel method for NO removal at low temperature. The developed Mn/TiO_2_ catalysts exhibited outstanding performance in the selective catalytic reduction (SCR) process, utilizing NH_3_ as the reductant, for NO removal at low temperatures. Furthermore, as the solid content increased, the catalyst’s activity at low temperatures gradually enhanced. Notably, the Mn/TiO_2_ (50%) catalyst demonstrates outstanding denitration performance, achieving a 96% NO conversion rate at 100 °C, and remained at 99% within the temperature range of 110–250 °C under a VHSV of 24,000 h^−1^, while maintaining high N_2_ selectivity and stability. Meanwhile, the correlation mechanism between performance test results and the increase in solid content was discovered through characterization testing: as the solid content increased, the catalyst’s specific surface area, surface Mn^4+^ concentration, and chemisorbed oxygen content all increased, positively impacting the SCR-NH_3_ low-temperature activity of the catalyst. Additionally, as the solid content of the catalyst increased, the number of acid sites on the catalyst surface and the reduction performance of the catalyst also increased, thereby enhancing the SCR-NH_3_ reaction activity at low temperatures. Furthermore, NH_3_ at the Lewis acidic sites and NH^4+^ at the Bronsted acidic sites of the catalyst were capable of reacting with NO. Conversely, NO and NO_2_ adsorbed on the catalyst, along with bidentate and monodentate nitrates, were unable to react with NH_3_ at low temperatures. Consequently, the developed catalyst’s low-temperature catalytic reaction mechanism aligns with the E-R mechanism.

## Figures and Tables

**Table 1:** The data related to SSA and pore size of catalysts.

Catalyst Sample	S_BET_ (m^2^/g)	Pore Volume (cm^3^/g)	Average Pore Diameter (nm)
Mn/TiO_2_ (20%)	146.44	0.2908	6.15
Mn/TiO_2_ (30%)	158.76	0.2613	5.03
Mn/TiO_2_ (40%)	152.77	0.2458	4.89
Mn/TiO_2_ (50%)	165.34	0.2365	4.38

**Table 2:** Surface atom concentration and peak area of catalysts.

Catalyst Sample	Mn^4+^/Mn^n+^ (%)	O_α_/(O_α_ + O_β_) (%)	H_2_-TPR Peak Area (%)	NH_3_-TPD Peak Area (%)
Mn/TiO_2_ (20%)	35.24	22.77	83.21	76.28
Mn/TiO_2_ (30%)	42.07	31.06	95.87	78.57
Mn/TiO_2_ (40%)	44.55	29.87	97.44	84.28
Mn/TiO_2_ (50%)	45.40	31.41	100.00	100.00

## Data Availability

The data presented in this study are available on request from the corresponding author.
